# Complete Mitochondrial Genome of *Niphon spinosus* (Perciformes: Niphonidae): Genome Characterization and Phylogenetic Analysis

**DOI:** 10.3390/biom15010052

**Published:** 2025-01-02

**Authors:** Maheshkumar Prakash Patil, Jong-Oh Kim, Seung Hyun Yoo, Jiyoung Shin, Ji-Young Yang, Kyunghoi Kim, Gun-Do Kim

**Affiliations:** 1 Industry-University Cooperation Foundation, Pukyong National University, 45 Yongso-ro, Nam-Gu, Busan 48513, Republic of Korea; 2 Department of Microbiology, Pukyong National University, 45 Yongso-ro, Nam-Gu, Busan 48513, Republic of Korea; 3 School of Marine and Fisheries Life Science, Pukyong National University, 45 Yongso-ro, Nam-Gu, Busan 48513, Republic of Korea; 4 Institute of Food Science, Pukyong National University, 45 Yongso-ro, Nam-Gu, Busan 48513, Republic of Korea; 5 Department of Food Science and Technology, Pukyong National University, 45 Yongso-ro, Nam-Gu, Busan 48513, Republic of Korea; 6 Department of Ocean Engineering, Pukyong National University, 45 Yongso-ro, Nam-Gu, Busan 48513, Republic of Korea

**Keywords:** *Niphon spinosus*, Percoidei, Perciformes, mitogenome, phylogenetic analysis, sawedged perch, marine fish

## Abstract

The species *Niphon spinosus* (Cuvier, 1829) is the only representative of the family Niphonidae and the genus *Niphon*, and its taxonomic history is complicated; it is still unclear in a phylogenetic sense. In this study, we report the complete mitochondrial genome of *N. spinosus* (OP391482), which was determined to be 16,503 bp long with biased A + T contents (53.8%) using next-generation technology. The typical set of 13 protein-coding genes (PCGs), 2 rRNA genes, 22 tRNA genes, and one control region (D-loop) are included in the mitochondrial genome. The H-strand encoded 28 genes (14 tRNA, 2 rRNA, and 12 PCGs), and D-loop, whereas the L-strand encoded the remaining 9 genes (8 tRNA and *ND6*). Its nucleotide composition, gene arrangement, codon usage patterns, and tRNA secondary structures are identical with other members of the Percoidei suborder. Furthermore, we reconstructed phylogenetic trees based on the 13 PCGs. The resulting phylogenetic trees showed *N. spinosus* placing as a separate lineage within the family Niphonidae, its close relationship to *Trachinus draco* (Trachinidae), and the clustering of major subfamilies like Luciopercinae and Percinae of the Percoidei suborder. These findings will contribute to future studies on the evolutionary history, population genetics, molecular taxonomy, and phylogeny of *N. spinosus* and related species.

## 1. Introduction

The sawedged perch, *Niphon spinosus* Cuvier, 1828, is a bony fish from the family Niphonidae, suborder Percoidei, and order Perciformes [1,2]. It is the only species in the Niphonidae family and *Niphon* genus. It dwells in the water at depths of up to 200 m and is found around the shores of East and Southeast Asia, including Korea, the Philippines, Japan, and China [3,4]. This marine ray-finned species dwells mostly on rock reefs and in inshore waters with rocky sea bottoms, and it can grow up to 1 m in length and weigh up to 11 kg [2].

*N. spinosus* was reclassified from Serranidae to the newly defined family Niphonidae after extensive morphological and phylogenetic analysis revealed considerable differences from other Serranidae members. In the past, Serranidae was used to describe a wide range of perch-like fish, including species with doubtful connections. However, Johnson [3] showed that *N. spinosus* has primitive features that make it different from other serranids. For example, its dorsal fin and pterygiophore structures, vertebral counts, and other skeletal characteristics are all unique. Subsequent research indicated that *N. spinosus* did not fit into these subgroups, needing its own family, Niphonidae, to properly represent its evolutionary history and morphological uniqueness [5].

The identification and classification of fish based on morphological features are not easy due to the high degree of similarities across several species. One such example is the sawedged perch (*N. spinosus*, Korean name: dageumbari), which is well known and popular among raw fish customers in the Republic of Korea. Although it lives in southern coastal seas, especially surrounding Jeju Island in Korea, its population is very limited. *N. spinosus* has morphological features that are similar to those of other species, such as the longtooth grouper (*Epinephelus bruneus*) and the convict grouper (*Epinephelus septemfasciatus*). These characteristics include body color, mouth shape, and the presence of body stripes, and these features gradually fade as they become adults, making them difficult to observe. Because of these difficulties, there have been several cases of misidentification and even mislabeling. As a result, molecular identification approaches, especially analysis of the 16S rRNA gene, provide reliable alternatives for accurate species identification [4]. The complete mitochondrial genome of *N. spinosus* has not been reported. Molecular studies using a complete mitochondrial genome or mitochondrial genes are widely accepted as more effective for resolving phylogenetic relationships than morphological features [6,7]. The complete mitochondrial genome of *N. spinosus* may help identify the species and establish its phylogenetic connections in the Perciformes order and also help to resolve taxonomic difficulties and improve our knowledge of Perciformes species evolution [8].

In this study, we report the first complete mitochondrial genome of *N. spinosus*, identify genomic characteristics, and perform phylogenetic analysis. The data generated in this study will be useful for phylogenetic analysis, species identification, population genetics, and evolutionary studies of Perciformes species.

## 2. Materials and Methods

### 2.1. Sample Collection and Genomic DNA Isolation

**Figure 1:**
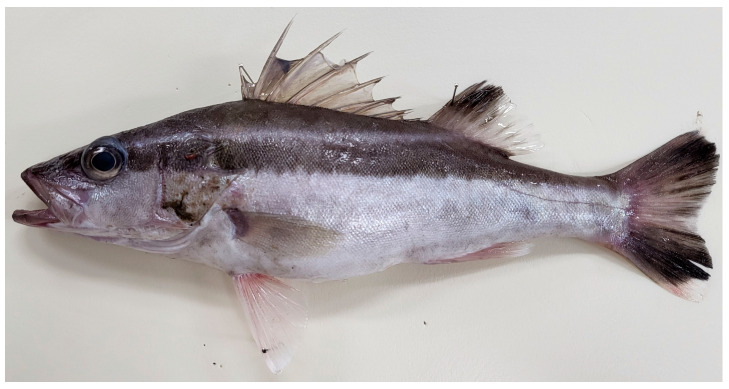
A sample image of *Niphon spinosus* (Photo by Dr. Ji-Young Yang), collected from the coast of Busan, Republic of Korea. It has an elongated, laterally compressed body with a pointed snout and large mouth. The sides are silver-gray and change to a darker shade; there are two dark brown stripes on the body, and there is a slightly convex area between the eyes.

*N. spinosus* specimens were collected from the coastal waters of the East Sea near Busan, Republic of Korea (35°1′58.09″ N, 129°1′54.93″ E) and deposited at the Department of Marine Biology, Pukyong National University, Busan, Republic of Korea under voucher number MFDS-FBA07 (Prof. Ji-Young Yang; jyyang@pknu.ac.kr) (Figure 1). Total genomic DNA was extracted from the muscle tissue samples using a DNeasy Blood and Tissue Kit (Qiagen, Germany). The quality and concentration of DNA were checked using a NANODROP D1000 spectrophotometer (Thermo Fisher Scientific, Waltham, MA, USA), after which the samples were stored at −4 °C until further analysis was performed.

### 2.2. Whole Genome Sequencing

The complete mitochondrial genome of *N. spinosus* underwent sequencing using Illumina Platform at Macrogen Company (https://dna.macrogen.com/) in Daejeon, Republic of Korea. DNA libraries were prepared using the TruSeq Nano DNA Kit (Illumina, Inc., San Diego, CA, USA) according to the manufacturer’s instructions, with a target insert size of 350 bp. Paired-end sequencing (2 × 150 bp) was performed by Macrogen on the Illumina HiSeq 2500 system.

### 2.3. Mitochondrial Genome Assembly

Low-quality reads (>20 Q20 and >30 Q30) and adapter sequences were removed using Trimmomatic [9], to minimize analysis biases. In the *N. spinosus* library, 17,613,260 total raw reads were generated with the following quality metrics: a GC content of 40.0%, Q20 at 91.7%, and Q30 at 82.6%. After filtering, 9,801,624 high-quality reads remained, with a GC content of 40.0%, Q20 at 98.5%, and Q30 at 93.9%. Sequencing quality was checked using FastQC v0.11.5 (Babraham Institute, Bioinformatics, http://www.bioinformatics.babraham.ac.uk/projects/fastqc, accessed on 1 July 2022) [10]. High-quality paired-end reads were randomly sampled for de novo assembly of the mitochondrial genome using NOVOPlasty v4.2.1 [11] with a *k*-mer size of 33 [12]. Contigs containing mitochondrial sequences were identified through BLAST analysis against the NCBI database (https://blast.ncbi.nlm.nih.gov/, accessed on 1 July 2022). The mitochondrial genome was annotated utilizing the MITOS (http://mitos.bioinf.uni-leipzig.de/index.py, accessed 1 August 2022) [13] to confirm the boundaries and orientation of each gene and MitoFish (http://mitofish.aori.u-tokyo.ac.jp/, accessed 1 August 2022) [14] pipelines for the validation of fish mitochondrial genome annotation. Predicted open reading frames (ORFs) underwent manual examination, and final annotations were confirmed using ORFfinder (https://www.ncbi.nlm.nih.gov/orffinder/, accessed 1 August 2022). Protein-coding genes (PCGs) were validated through a BLAST homology search against previously reported mitogenomes of Perciformes [15]. Transfer RNAs (tRNAs) were identified using tRNAscan-SE 2.0 (http://lowelab.ucsc.edu/tRNAscan-SE/, accessed 1 August 2024) with default vertebrate mitochondrial parameters [16], and their secondary structures were verified using ARWEN [17]. The assembled contig was further validated using BlastN [18] and by comparing its size with known Perciformes mitogenomes to ensure accuracy and completeness.

A visual representation of the *N. spinosus* mitogenome was created by the Chloroplot online program [19] available on the MitoFish online platform (https://mitofish.aori.u-tokyo.ac.jp/annotation/input/, accessed 1 October 2024). The nucleotide compositions of the mitochondrial genome and the relative synonymous codon usage (RSCU) of the PCGs were estimated using MEGA11 [20]. For RSCU analysis, stop codons (incomplete or complete) of the PCGs were removed before analysis to avoid errors formation in triplets, to maintain consistency in coding regions, and to focus on synonymous codons. Skew analysis was performed using the formulas AT-skew = (A − T)/(A + T) and GC-skew = (G − C)/(G + C) [21]. Intergenic spacers and overlapping gene regions were calculated manually. The tandem repeats were determined using the online Tandem Repeats Finder program V4.09 (https://tandem.bu.edu/trf/, accessed 1 October 2024) [22].

### 2.4. Phylogenetic Tree Construction

To determine the evolutionary relationship of *N. spinosus* with Percoidei species, phylogenetic analysis was performed using 51 Percoidei species from different families (Table S1). This includes representatives from Percidae (41 species), Percinae (4 species), Luciopercinae (3 species), Trachinidae (2 species), and Niphonidae (1 species). The *Chaetodon nippon* (ON843632) mitochondrial genome from the order Chaetodontiformes was chosen as the outgroup taxon. The NCBI database was the source of all sequence downloads. Concatenated nucleotide sequences from the 13 PCGs in the following order served as the basis for the phylogenetic analysis: *ND1*, *ND2*, *COX1*, *COX2*, *ATP8*, *ATP6*, *COX3*, *ND3*, *ND4L*, *ND4*, *ND5*, *ND6*, and *CYTB*. The multiple sequences alignment was performed using the MAFFT v7.0 online platform [23]. The best-fit model for each partition was determined using IQ-TREE’s ModelFinder function (partition-specific model selection, Table S2) and with the lowest Bayesian Information Criterion (BIC) scores [24,25]. The IQ-TREE online server and PhyML v3.0 were used to construct the maximum likelihood (ML) phylogenetic tree, which included 1000 bootstrap repetitions [26]. The Bayesian (BA) phylogenetic tree was produced using MrBayes 3.1.2, adopting the GTR + I (nst = 6) model with one cold and three hot Metropolis-coupled Markov chain Monte Carlo (MCMC) runs. The study was run for 10,000,000 generations, with samples taken every 100 generations and the first 25% eliminated as burn-in [27]. The generated phylogenetic trees were displayed using the iTOL v7 web server (https://itol.embl.de/login.cgi, visited 16 December 2024) [28].

## 3. Results and Discussion

### 3.1. Genome Structure and Nucleotide Composition

**Figure 2:**
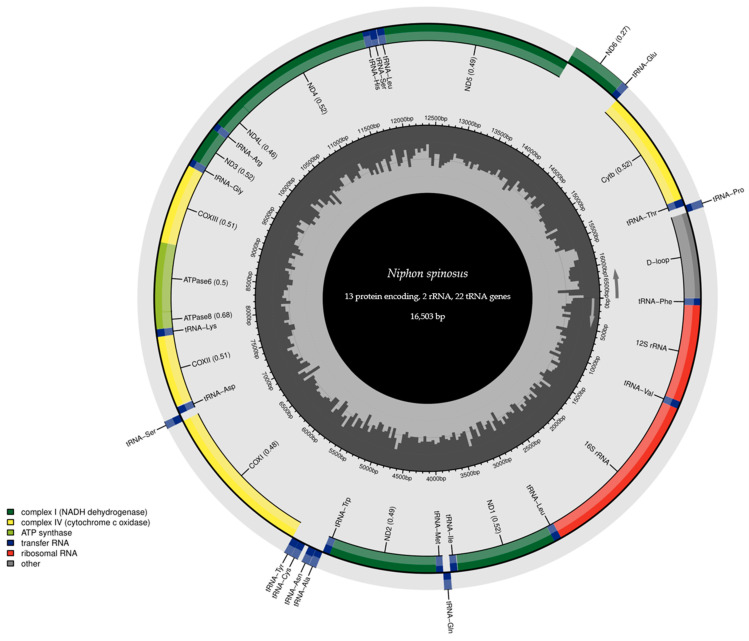
Gene map of the complete mitochondrial genome of *Niphon spinosus* (OP391482). Genes located on the heavy strand are displayed on the inner ring of the circle, while genes on the light strand are shown on the outer ring. The innermost circle represents the GC content, with darker lines indicating regions of higher GC content.

The complete mitochondrial genome sequence of *N. spinosus* has 16,503 bp length and is composed of 37 genes and one control region, including 2 rRNA genes (*16S rRNA*, *12S rRNA*), 13 PCGs (*ND1* to *ND6*, *ND4L*, *COX1* to *COX3*, *ATP6*, *ATP8*, *CYTB*), 22 tRNA genes, and one D-loop region (Table 1). The *ND6* gene and eight tRNA genes (*tRNA-Glu*, *tRNA-Pro*, *tRNA-Gln*, *tRNA-Ala*, *tRNA-Asn*, *tRNA-Cys*, *tRNA-Tyr*, *tRNA-Ser*) were encoded on the L-strand, while the remaining genes were encoded on the H-strand (Figure 2). In *N. spinosus*, ten intergenic spacer regions and six overlapping regions, totaling 67 and 24 bp, respectively, were found. The gene structure and arrangement of these species exhibited no instances of gene rearrangement. Mitochondrial gene rearrangements are often associated with genetic variants, physiological properties, molecular mechanisms, lifecycle features, or genomic evolution processes [29,30]. The mitochondrial genome sequence of *N. spinosus* was submitted to GenBank and is available under the accession number OP391482. The mitochondrial genome length of *N. spinosus* is shorter compared to those of the species in the Percoidei suborder, which ranges from 16,536 bp (*Perca schrenkii*) to 16,924 bp (*Echiichthys vipera*). However, the total number of genes is similar to those of the species in the Percoidei suborder [31,32,33,34].

The *N. spinosus* mitochondrial genome revealed the following nucleotide composition: A = 27.8%, T = 26.0%, G = 16.9%, and C = 29.4%, with a total A + T content of 53.8%, comparable to those of other Percoidei species. Skew analysis revealed a positive AT-skew value (0.034), suggesting a greater presence of A than T, and a negative GC-skew value (−0.271), indicating more C than G. Other Trachinidae species, such as *E. vipera* and *Trachinus draco*, as well as the Percidae species *Sander lucioperca* [34], show similar positive AT-skew and negative GC-skew patterns. Overall, the mitochondrial genome of *N. spinosus* is comparable to those of other Percoidei species in terms of genome length, nucleotide content, gene organization patterns, and skewness.

### 3.2. Protein-Coding Genes

The 13 PCGs in *N. spinosus* comprise a total length of 11,428 bp, accounting for 69.3% of the complete mitochondrial genome. Twelve genes were situated on the H-strand, whereas only the *ND6* gene was located on the L-strand. Among the PCGs, *ATP6* was the shortest gene at 168 bp, while *ND5* was the longest at 1839 bp (Table 1). Most PCGs initiate with the ATG codon, with the exception of the *COX1* gene, which starts with the GTG codon. The utilization of GTG for *COX1* is characteristic of bony fish and aligns with findings from other studies [35,36]. TAA serves as a stop codon for the *COX3*, *ATP8*, and *ND4L* genes, whereas TAG is utilized by the *ND1*, *ND5*, and *ND6* genes. Seven PCGs (*ND2*, *COX2*, *ATP6*, *COX3*, *ND3*, *ND4*, *CYTB*) exhibit incomplete stop codons. Incomplete stop codons are common in fish mitochondrial genomes and can be completed through the addition of poly A tails during RNA processing [37,38]. The PCG characteristics of *N. spinosus* align with those of other species within the Percoidei suborder [31,32,33,34].

The A + T content of the 13 PCGs in *N. spinosus* was 53.0%, with values ranging from 50.8% for *ND4L* to 56.0% for *ATP8*. Additionally, base skews were calculated to assess the extent of base bias among all the PCGs. The trends of AT-skew and GC-skew values across all 13 PCGs of *N. spinosus* are given in Table S3. Twelve of the thirteen PCGs exhibited significant negative GC skewness, with an exception noted in the *ND6* region, in line with findings in most Perciformes fish [31,32,33,34,35,36,39].

### 3.3. Relative Synonymous Codon Usage

**Figure 3:**
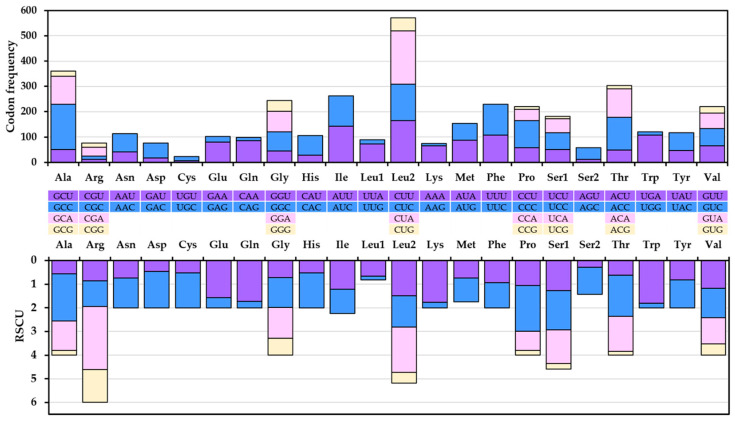
Codon frequency and relative synonymous codon usage (RSCU) of the mitochondrial PCGs of *Niphon spinosus* (OP391482).

*N. spinosus* mitochondrial PCG codon usage and RSCU analysis results are presented in Figure 3. The PCGs code for a total of 3800 amino acids. The most frequently utilized codons were those for leucine (17.4%), alanine (9.5%), and threonine (8.0%), with cysteine as the least used (0.6%). The amino acid distribution and relative frequency in *N. spinosus* were similar to those found in the mitochondrial genomes of other Percoidei species (Table S4). RSCU values help to identify codon use bias. A number greater than one indicates a positive bias, a number less than one indicates a negative bias, and one indicates no bias [40]. In this study, the codons CGA (R), GCC (A), CCC (P), and CUA (L) had a significant bias, with RSCU values of 2.65, 2.0, 1.94, and 1.91, respectively. Whereas the AUG (M) is the only codon with an RSCU value of 1.0, similar values were also observed in other species of the families Percinae (except *Gymnocephalus cernua*), Luciopercinae, and Trachinidae, as used in this study (Table S4). The codon frequency and RSCU values in *N. spinosus* were similar to those in other Percoidei species. Understanding codon use patterns is important for exploring gene expression and evolutionary processes [41].

### 3.4. Ribosomal RNA and Transfer RNA Genes

*N. spinosus*, like other fish, also possessed two rRNAs genes with a length of 2642 bp (16.0% of the total mitochondrial genome). The large (*16S rRNA*) ribosomal gene was 1695 bp long and was located between the *tRNA-Val* and *tRNA-Leu* genes, while the small ribosomal gene (*12S rRNA*) was 947 bp long and located between the *tRNA-Phe* and *tRNA-Val* genes (Table 1). Both rRNA genes were located on the H-strand, which were separated by the *tRNA-Val* gene, a characteristic that is commonly observed in other Percoidei species [31,32,33,34]. The number of RNA genes in *N. spinosus* is similar to that of other Percoidei species used in this study (Table S1).

**Figure 4:**
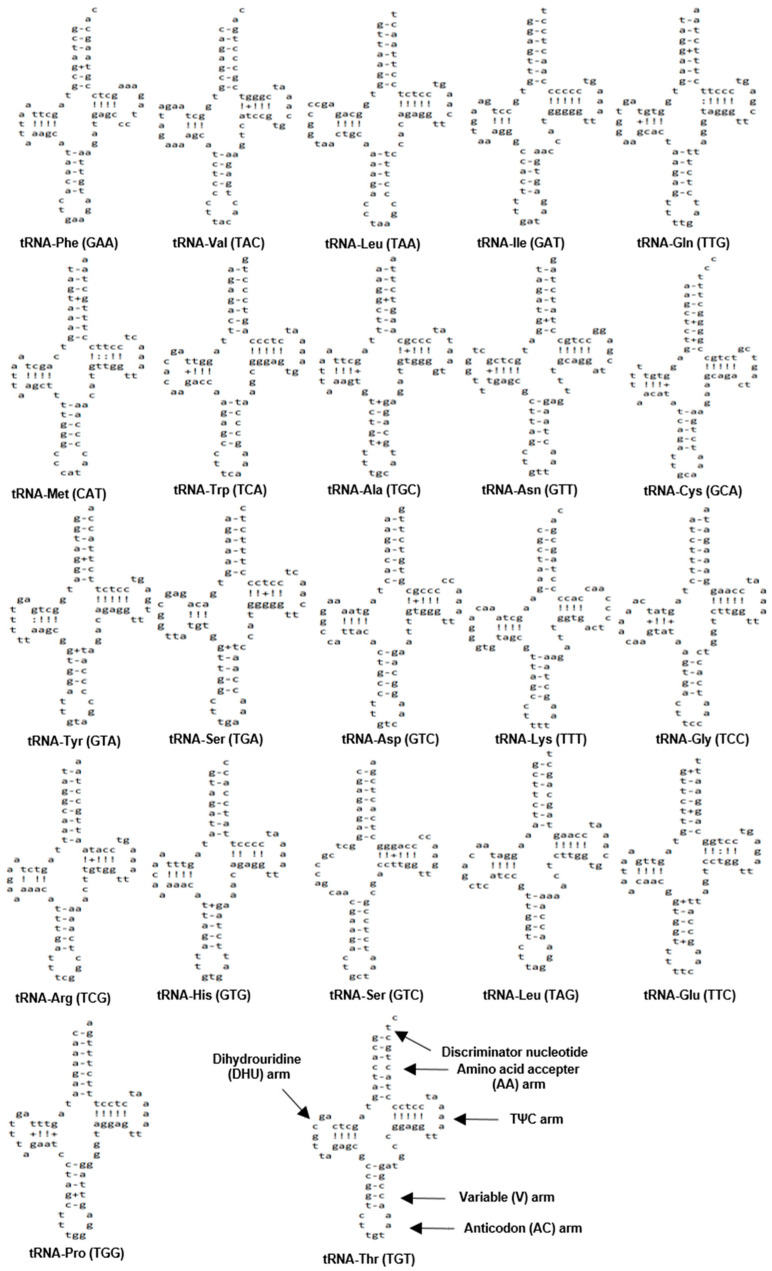
Inferred secondary structure of the 22 tRNA genes in the *Niphon spinosus* (OP391482) mitochondrial genome exhibit structural variations. Watson–Crick and Wobble base pairing (GT bonds) are illustrated as “−”, and “+”, respectively.

The mitochondrial genome of *N. spinosus* contains the complete set of 22 tRNA genes, individually ranging in size from 68 bp to 74 bp (Table 1). These genes make up 25.4% (4196 bp) of the entire mitochondrial genome. To be more specific, 15 of these tRNA genes were encoded on the H-strand and 7 on the L-strand (Table 1, Figure 2), which is similar with the tRNA gene distribution observed in other Percoidei species [31,32,33,34]. The anticodons of all the tRNAs in the mitochondrial genome of *N. spinosus* were identical to those in most of the Percoidei species. In general, each codon corresponds to a particular anticodon. However, serine was encoded by two distinct anticodons (UGA, GCU), and leucine was encoded by UAA and UAG in *N. spinosus*. The mitochondrial genomes of Perciformes fish are frequently characterized by the presence of multiple tRNAs that recognize distinct anticodons [35,36]. The secondary structures of tRNAs are shown in Figure 4. All the tRNA genes were folded into the canonical cloverleaf secondary structures, with the exception of *tRNA-Ser* (GCT), which is missing the dihydrouridine (DHU) arm, a characteristic frequently observed in bony fish [42]. Wobble base pairings were identified in 17 tRNAs of *N. spinosus*, with the most notable occurrences found in *tRNA-Ala* and *tRNA-Glu*. Specifically, these pairings were found in the AA arm of eight tRNAs, the TΨC arm of six tRNAs, the variable arm of four tRNAs, and the DHU arm of seven tRNAs. Wobble pairing, an important feature of RNA structure, often replaces AT or GC base pairs due to its thermodynamic stability. Additionally, homogeneity in the anticodons of all 22 tRNAs was observed in *N. spinosus*. These characteristics play key roles in various biological processes [37,41,43], highlighting the importance of comprehensive studies of tRNAs to better understand the structural and functional attributes of fish mitogenomes [38].

### 3.5. Characteristics of Control Region

The D-loop (control region) of *N. spinosus* spans 836 bp (Table S1), accounting for 5.1% of the complete mitochondrial genome. The nucleotide composition includes A = 290 (34.7%), T = 250 (29.9%), G = 129 (15.4%), and C = 167 (20.0%). This region exhibits an A + T bias of 64.6%, with a positive AT-skew (0.074) and a negative GC-skew (−0.128). Encoded on the H-strand, the control region is located between the *tRNA-Pro* and *tRNA-Phe* genes (Figure 2), consistent with those of other species within Percoidei [31,32,33,34]. However, unlike other fish species, the *N. spinosus* control region does not contain tandem repeats.

### 3.6. Phylogenetic Relationships

**Figure 5:**
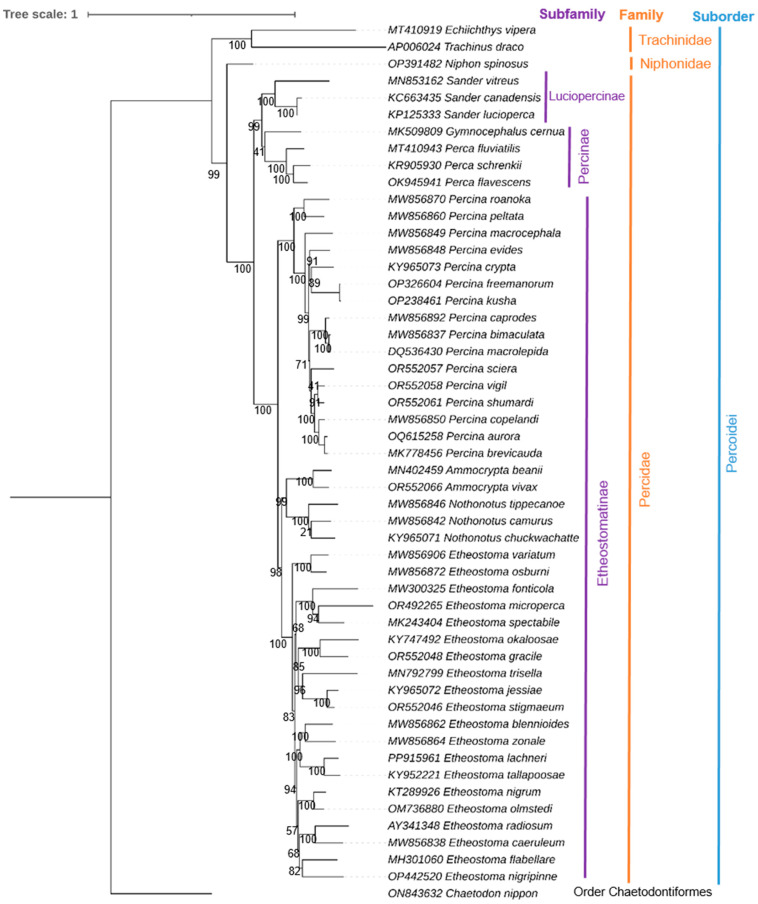
Maximum Likelihood (ML) topology built using the concatenated 13 PCGs, effectively distinguishing *Niphon spinosus* from other species of the Percoidei order. The cladogram also offers perceptions into the evolutionary relations across diverse taxonomic levels (subfamily and family) within the Percoidei suborder. ML bootstrap support values are annotated at each node, indicating the statistical support for individual branches in the topology.

The ML and BA topologies, constructed using the concatenated 13 PCGs, distinctly separate all Percoidei species, elucidating their evolutionary relationship (Figure 5 and Figure S1). Species from various taxonomic lineages, spanning both family and subfamily levels, display distinct monophyletic clustering patterns. The ML tree results demonstrate the evolutionary relationships of *N. spinosus* (family Niphonidae) within the suborder Percoidei and position it as a distinct lineage, closely related to *Trachinus draco* (family Trachinidae). Other subfamilies, such as Luciopercinae (*Sander* species) and Percinae (*Gymnocephalus* and *Perca* species), form strongly supported clades, indicating close genetic relationships within these groups. The largest clade, Etheostomatinae, exhibits complex branching patterns and includes species from genera such as *Percina*, *Ammocrypta*, *Nothonotus*, and *Etheostoma*. The formation of a closely linked group of these species indicate that they had a recent common ancestor [31,32,33,34]. *C. nippon* (order Chaetodontiformes) serves as the outgroup, confirming the phylogenetic distinctiveness of Percoidei. High bootstrap values at key nodes reflect strong statistical support for the tree’s topology and evolutionary relationships.

Both the ML (Figure 5) and BA (Figure S1) phylogenetic trees share significant similarities in the overall topology and relationships among the taxa. Key similarities include the distinct placement of *N. spinosus* as a separate lineage within the family Niphonidae, its close relationship to *Trachinus draco* (Trachinidae), and the clustering of major subfamilies like Luciopercinae and Percinae into well-supported clades. Both methods also confirm *C. nippon* as an outgroup, reinforcing the evolutionary divergence of Percoidei. High statistical support (posterior probabilities in BA and bootstrap values in ML) at critical nodes further validates the consistency between the two approaches.

## 4. Conclusions

This study reports the first complete mitochondrial genome of *N. spinosus*, the only species in the Niphonidae family and *Niphon* genus. The mitochondrial features of *N. spinosus* are in line with Percoidei species in terms of genome length, nucleotide composition, biasness, skewness, and genes organization. Phylogenetic relationships were established using ML and BA trees based on the concatenated 13 PCGs to reveal the evolutionary relationships among Percoidei species. The genomic data generated in this study will be very useful for species identification, phylogenetic investigations, and population genetics. There is a need for more mitochondrial genome research across Perciformes species to better understand evolutionary connections within the order. This study’s focus on the mitochondrial genome suggests that future research including nuclear genome-wide analyses could significantly enhance the resolution of taxonomic confusion within the fish family.

## Figures and Tables

**Table 1:** Genomic characteristics of the complete mitochondrial genome of *Niphon spinosus* (OP391482). In intergenic nucleotides (IN), positive numbers indicate overlap, whereas negative values denote gaps between adjacent genes. Genes encoded on the heavy and light strands are indicated by the letters H and L in the strand column, respectively.

Gene	Position		Size (bp)	Strand	IN	Codon		Anti-Codon	Amino Acids
	Start	End				Start	Stop		
*tRNA^Phe^*	1	68	68	H	0	-	-	GAA	-
*12S rRNA*	69	1015	947	H	0	-	-	-	-
*tRNA^Val^*	1016	1087	72	H	0	-	-	TAC	-
*16S rRNA*	1088	2782	1695	H	0	-	-	-	-
*tRNA^Leu^*	2783	2856	74	H	0	-	-	TAA	-
*ND1*	2857	3831	975	H	0	ATG	TAG	-	324
*tRNA^Ile^*	3836	3905	70	H	4	-	-	GAT	-
*tRNA^Gln^*	3905	3975	71	L	−1	-	-	TTG	-
*tRNA^Met^*	3975	4043	69	H	−1	-	-	CAT	-
*ND2*	4044	5089	1046	H	0	ATG	TA-	-	348
*tRNA^Trp^*	5090	5160	71	H	0	-	-	TCA	-
*tRNA^Ala^*	5162	5230	69	L	1	-	-	TGC	-
*tRNA^Asn^*	5232	5304	73	L	1	-	-	GTT	-
*tRNA^Cys^*	5344	5411	68	L	39	-	-	GCA	-
*tRNA^Tyr^*	5412	5482	71	L	0	-	-	GTA	-
*COX1*	5484	7034	1551	H	1	GTG	TAA	-	516
*tRNA^Ser^*	7035	7105	71	L	0	-	-	TGA	-
*tRNA^Asp^*	7109	7179	71	H	3	-	-	GTC	-
*COX2*	7188	7878	691	H	8	ATG	T--	-	230
*tRNA^Lys^*	7879	7952	74	H	0	-	-	TTT	-
*ATP8*	7954	8121	168	H	1	ATG	TAA	-	55
*ATP6*	8112	8794	683	H	−10	ATG	TA-	-	227
*COX3*	8795	9579	785	H	0	ATG	TA-	-	261
*tRNA^Gly^*	9580	9651	72	H	0	-	-	TCC	-
*ND3*	9652	10,000	349	H	0	ATG	T--	-	116
*tRNA^Arg^*	10,001	10,069	69	H	0	-	-	TCG	-
*ND4L*	10,070	10,366	297	H	0	ATG	TAA	-	98
*ND4*	10,360	11,740	1381	H	−7	ATG	T--	-	460
*tRNA^His^*	11,741	11,809	69	H	0	-	-	GTG	-
*tRNA^Ser^*	11,810	11,877	68	H	0	-	-	GCT	-
*tRNA^Leu^*	11,882	11,954	73	H	4	-	-	TAG	-
*ND5*	11,955	13,793	1839	H	0	ATG	TAG	-	612
*ND6*	13,790	14,311	522	L	−4	ATG	TAG	-	173
*tRNA^Glu^*	14,312	14,380	69	L	0	-	-	TTC	-
*CYTB*	14,386	15,526	1141	H	5	ATG	T--	-	380
*tRNA^Thr^*	15,527	15,598	72	H	0	-	-	TGT	-
*tRNA^Pro^*	15,598	15,667	70	L	−1	-	-	TGG	-
D-loop	15,668	16,503	836	H	0	-	-	-	-

## Data Availability

The genome sequence data that support the findings of this study are openly available in GenBank of NCBI at (https://www.ncbi.nlm.nih.gov/) under the accession no. OP391482. The associated BioProject, BioSample, and SRA numbers are PRJNA903969, SAMN31823545, and SRR22366792, respectively.

## Supplementary Materials

The following supporting information can be downloaded at https://www.mdpi.com/article/10.3390/biom15010052/s1, Table S1: a list of mitochondrial genomes from Percoidei species used in this study, with Chaetodon nippon included as the outgroup; Table S2: a list of models used in the partitioned analysis method for constructing phylogenetic trees based on various protein-coding genes (PCGs) of 53 fish species; Table S3: nucleotide composition of the mitochondrial PCGs of *Niphon spinosus* (OP391482); Table S4: codon frequencies and relative synonymous codon usage (RSCU) of the mitochondrial PCGs of fish species of Percoidei; Figure S1: the Bayesian phylogeny constructed from the 13 concatenated PCGs clearly distinguishes *Niphon spinosus* from other species of Percoidei. Bayesian posterior probability support values are indicated at each node, reflecting the statistical support for each branching point in the tree.
