# Supplementary material for: Complete Mitochondrial Genome of Niphon spinosus (Perciformes: Niphonidae): Genome Characterization and Phylogenetic Analysis
Source: Biomolecules. 2025 Jan 2;15(1):52. doi: 10.3390/biom15010052 (PMC11764044; doi:10.3390/biom15010052)
Supplement: Supplementary file 1 [file biomolecules-15-00052-s001.zip › biomolecules-3357596-supplementary.pdf]

SUPPLIMENTARY DATA

Table S1. List of mitochondrial genomes from Percoidei species used in this study, with Chaetodon nippon included as the outgroup

Family (subfamily)	Name	Accession No.	Length (bp)		Genes		
			Total	D-loop	rRNA	tRNA	PCG
Niphonidae	*Niphon sphinosus*	OP391482	16,503	836	2	22	13
Trachinidae	*Echiichthys vipera*	MT410919	16,924	981	2	22	13
	Trachinus draco	AP006024	16,810	850	2	22	13
Percidae (Etheostomatinae)	*Etheostoma olmstedi*	OM736880	16,615	841	2	22	13
	Etheostoma tallapoosae	KY952221	16,592	932	2	22	13
	Etheostoma microperca	OR492265	16,690	608	2	22	13
	Etheostoma spectabile	MK243404	16,539	882	2	22	13
	Etheostoma gracile	OR552048	16,579	920	2	22	13
	Etheostoma osburni	MW856872	16,699	1,039	2	22	13
	Etheostoma radiosum	AY341348	16,600	938	2	22	13
	Etheostoma nigripinne	OP442520	16,662	1,002	2	22	13
	Etheostoma okaloosae	KY747492	16,616	829	2	22	13
	Etheostoma flabellare	MH301060	16,686	1,030	2	22	13
	Etheostoma fonticola	MW300325	16,662	1,002	2	22	13
	Etheostoma jessiae	KY965072	16,600	943	2	22	13
	Etheostoma lachneri	PP915961	16,598	939	2	22	13
	Etheostoma variatum	MW856906	16,592	933	2	22	13
	Etheostoma caeruleum	MW856838	16,589	929	2	22	13
	Etheostoma zonale	MW856864	16,586	931	2	22	13
	Etheostoma nigrum	KT289926	16,579	920	2	22	13
	Etheostoma trisella	MN792799	16,572	915	2	22	13
	Etheostoma blennioides	MW856862	16,641	980	2	22	13
	Etheostoma stigmaeum	OR552046	16,557	900	2	22	13
	Percina brevicauda	MK778456	16,608	943	2	22	13
	Percina crypta	KY965073	16,605	941	2	22	13
	Percina peltata	MW856860	16,624	964	2	22	13
	Percina caprodes	MW856892	16,624	964	2	22	13
	Percina copelandi	MW856850	16,607	946	2	22	13
	Percina bimaculata	MW856837	16,604	944	2	22	13
	Percina macrolepida	DQ536430	16,602	941	2	22	13
	Percina macrocephala	MW856849	16,591	934	2	22	13
	Percina evides	MW856848	16,572	915	2	22	13
	Percina roanoka	MW856870	16,702	1,044	2	22	13
	Percina aurora	OQ615258	16,646	943	2	22	13
	Percina shumardi	OR552061	16,627	967	2	22	13
	Percina kusha	OP238461	16,629	965	2	22	13
	Percina vigil	OR552058	16,615	954	2	22	13
	Percina sciera	OR552057	16,613	953	2	22	13
	Percina freemanorum	OP326604	16,578	917	2	22	13
	Ammocrypta beanii	MN402459	16,682	1,024	2	22	13
	Ammocrypta vivax	OR552066	16,655	1,002	2	22	13
	Nothonotus camurus	MW856842	16,639	978	2	22	13
	Nothonotus chuckwachatte	KY965071	16,603	942	2	22	13

Family (subfamily)	Name	Accession No.	Length (bp) Total	D-loop	Genes rRNA	tRNA	PCG
	Nothonotus tippecanoe	MW856846	16,599	941	2	22	13
Percidae (Lucioperci-nae)	*Sander lucioperca*	KP125333	16,542	891	2	22	13
	Sander canadensis	KC663435	16,542	892	2	22	13
	Sander vitreus	MN853162	16,892	1,187	2	22	13
Percidae (Percinae)	*Perca fluviatilis*	MT410943	16,539	-	2	22	13
	Perca flavescens	OK945941	16,540	587	2	22	13
	Perca schrenkii	KR905930	16,536	872	2	22	13
	Gymnocephalus cernua	MK509809	16,677	1,014	2	22	13
Chaetodontidae	*Chaetodon nippon*	ON843632	16,507	851	2	22	13

Table S2. List of models used in the partitioned analysis method for constructing phylogenetic trees based on various protein-coding genes (PCGs) of 53 fishes' species

PCG	Part	Site	Best-fit model
ND1	Part 1	975	GTR+F+I+G4
ND2	Part 2	1046	TIM3+F+I+G4
COX1	Part 3	1551	TIM2+F+I+G4
COX2	Part 4	691	TPM2u+F+I+G4
ATP8	Part 5	168	TN+F+G4
ATP6	Part 6	683	TIM2+F+I+G4
COX3	Part 7	785	TIM2+F+I+G4
ND3	Part 8	349	TN+F+I+G4
ND4L	Part 9	297	TIM2+F+I+G4
ND4	Part 10	1381	GTR+F+I+G4
ND5	Part 11	1839	TIM2+F+I+G4
ND6	Part 12	522	TIM3+F+I+G4
CYTB	Part 13	1141	GTR+F+I+G4

Table S3. Nucleotide composition of the mitochondrial PCGs of *Niphon spinosus* (OP391482)

PCG	Length	A		T		G		C		A+T		G+C		AT-	GC-
	(bp)	count	%	count	%	count	%	count	%	count	%	count	%	Skew	Skew
ND1	975	240	24.62	275	28.21	158	16.21	302	30.97	515	52.82	460	47.18	-0.0680	-0.3130
ND2	1046	283	27.06	257	24.57	129	12.33	377	36.04	540	51.63	506	48.37	0.0481	-0.4901
COX1	1551	364	23.47	458	29.53	291	18.76	438	28.24	822	53.00	729	47.00	-0.1144	-0.2016
COX2	691	195	28.22	189	27.35	111	16.06	196	28.36	384	55.57	307	44.43	0.0156	-0.2769
ATP8	168	53	31.55	41	24.40	17	10.12	57	33.93	94	55.95	74	44.05	0.1277	-0.5405
ATP6	683	171	25.04	200	29.28	90	13.18	222	32.50	371	54.32	312	45.68	-0.0782	-0.4231
COX3	785	195	24.84	223	28.41	135	17.20	232	29.55	418	53.25	367	46.75	-0.0670	-0.2643
ND3	349	69	19.77	106	30.37	57	16.33	117	33.52	175	50.14	174	49.86	-0.2114	-0.3448
ND4L	297	70	23.57	81	27.27	43	14.48	103	34.68	151	50.84	146	49.16	-0.0728	-0.4110
ND4	1381	366	26.50	370	26.79	210	15.21	435	31.50	736	53.29	645	46.71	-0.0054	-0.3488
ND5	1839	504	27.41	484	26.32	270	14.68	581	31.59	988	53.72	851	46.28	0.0202	-0.3655
ND6	522	88	16.86	188	36.02	167	31.99	79	15.13	276	52.87	246	47.13	-0.3623	0.3577
CYTB	1141	271	23.75	322	28.22	179	15.69	369	32.34	593	51.97	548	48.03	-0.0860	-0.3467
Total	11428	2869	25.11	3194	27.95	1857	16.25	3508	30.70	6063	53.05	5365	46.95	-0.0536	-0.3077

Table S4. Codon frequencies and relative synonymous codon usage (RSCU) of the mitochondrial PCGs of fish species of Percoidei
Note: * = the termination codon, A = Ala, F = Phe, C = Cys, D = Asp, N = Asn, E = Glu, Q = Gln, G = Gly, H = His, L = Leu, I = Ile, K = Lys, M = Met, P = Pro, R = Arg, S = Ser, T = Thr, V = Val, W = Trp, Y = Tyr

**Codon**	***Niphon spinosus* (OP391482)**		***Perca flavescens* (OK945941)**		***Perca fluviatilis* (MT410943)**		***Perca schrenkii* (KR905930)**		***Gymnocephalus cernua* (MK509809)**		***Sander canadensis* (KC663435)**		***Sander lucioperca* (KP125333)**		***Sander vitreus* (MN853162)**		***Echiichthys vipera* (MT410919)**		***Trachinus draco* (AP006024)**	
	**Count**	**RSCU**	**Count**	**RSCU**	**Count**	**RSCU**	**Count**	**RSCU**	**Count**	**RSCU**	**Count**	**RSCU**	**Count**	**RSCU**	**Count**	**RSCU**	**Count**	**RSCU**	**Count**	**RSCU**
UUU (F)	108	0.94	129	1.10	124	1.06	132	1.15	128	1.09	119	1.03	120	1.03	120	1.01	130	1.16	99	0.87
UUC (F)	122	1.06	105	0.90	111	0.94	98	0.85	107	0.91	113	0.97	114	0.97	117	0.99	95	0.84	129	1.13
UUA (L)	73	0.66	122	1.10	110	0.99	109	0.98	110	0.99	122	1.11	126	1.14	115	1.06	96	0.86	108	0.98
UUG (L)	17	0.15	15	0.13	21	0.19	25	0.23	9	0.08	11	0.10	12	0.11	15	0.14	18	0.16	16	0.15
UCU (S)	51	1.28	50	1.28	47	1.20	51	1.30	44	1.13	53	1.31	47	1.18	56	1.37	52	1.27	50	1.24
UCC (S)	66	1.66	60	1.53	67	1.17	57	1.45	70	1.79	73	1.81	78	1.96	73	1.79	70	1.71	81	2.01
UCA (S)	56	1.41	58	1.48	58	1.48	57	1.45	57	1.46	52	1.29	52	1.31	50	1.22	49	1.20	41	1.02
UCG (S)	9	0.23	12	0.31	8	0.20	115	0.38	9	0.23	9	0.22	8	0.20	7	0.17	11	0.27	9	0.22
UAU (Y)	47	0.81	54	0.96	56	0.98	55	0.97	52	0.92	56	0.98	57	1.01	51	0.89	58	0.93	51	0.84
UAC (Y)	69	1.19	58	1.04	58	1.02	58	1.03	61	1.08	58	1.02	56	0.99	64	1.11	67	1.07	71	1.16
UAA (*)	0	0	0	0	0	0	0	0	0	0	0	0	0	0	0	0	0	0	0	0
UAG (*)	0	0	0	0	0	0	0	0	0	0	0	0	0	0	0	0	0	0	0	0
UGU (C)	6	0.52	8	0.70	9	0.78	9	0.78	7	0.61	5	0.42	5	0.43	10	0.83	6	0.48	7	0.50
UGC (C)	17	1.48	15	1.30	14	1.22	14	1.22	16	1.39	19	1.58	18	1.57	14	1.17	19	1.52	21	1.50
UGA (W)	108	1.80	100	1.67	106	1.77	91	1.52	105	1.75	5	0.08	101	1.68	93	1.56	96	1.64	93	1.55
UGG (W)	12	0.20	20	0.33	14	0.23	29	0.48	15	0.25	116	1.92	19	0.32	26	0.44	21	0.36	27	0.45
CUU (L)	164	1.49	200	1.80	198	1.78	199	1.79	180	1.62	200	1.83	204	1.84	182	1.69	185	1.66	171	1.56
CUC (L)	145	1.32	124	1.12	119	1.07	118	1.06	139	1.25	111	1.01	111	1.00	128	1.19	113	1.01	151	1.38
CUA (L)	210	1.91	153	1.38	171	1.54	163	1.47	179	1.61	179	1.63	179	1.62	146	1.35	210	1.89	165	1.50
CUG (L)	52	0.47	53	0.48	48	0.43	52	0.47	51	0.46	34	0.31	33	0.30	62	0.57	46	0.41	47	0.43
CCU (P)	58	1.05	91	1.65	87	1.57	82	1.48	85	1.55	82	1.43	75	1.37	71	1.27	80	1.43	77	1.39
CCC (P)	107	1.94	83	1.50	83	1.50	84	1.52	83	1.51	91	1.59	93	1.70	102	1.82	100	1.79	99	1.79
CCA (P)	45	0.81	39	0.71	40	0.72	38	0.69	42	0.76	54	0.94	48	0.88	42	0.75	33	0.59	34	0.62
CCG (P)	11	0.20	8	0.14	11	0.20	17	0.31	10	0.18	2	0.03	3	0.05	9	0.16	10	0.18	11	0.20
CAU (H)	28	0.53	35	0.66	39	0.74	39	0.74	35	0.64	40	0.71	39	0.71	35	0.67	31	0.55	33	0.62
CAC (H)	78	1.47	71	1.34	66	1.26	67	1.26	74	1.36	72	1.29	71	1.29	70	1.33	82	1.45	74	1.38
CAA (Q)	85	1.73	80	1.62	84	1.66	77	1.56	82	1.71	84	1.75	85	1.73	72	1.53	83	1.73	78	1.59
CAG (Q)	13	0.27	19	0.38	17	0.34	22	0.44	14	0.29	12	0.25	13	0.27	22	0.47	13	0.27	20	0.41
CGU (R)	11	0.86	14	1.08	15	1.18	16	1.22	15	0.78	16	1.30	16	1.26	10	0.78	13	1.04	14	1.15
CGC (R)	14	1.09	15	1.15	13	1.03	13	0.99	11	0.57	12	0.97	13	1.03	17	1.32	14	1.12	19	1.56
CGA (R)	34	2.65	44	3.38	40	3.16	37	2.81	42	2.18	40	3.24	41	3.24	38	2.96	42	3.36	31	2.55

Codon	*Niphon spinosus* (OP391482)		*Perca flavescens* (OK945941)		*Perca fluviatilis* (MT410943)		*Perca schrenkii* (KR905930)		*Gymnocephalus cernua* (MK509809)		*Sander canadensis* (KC663435)		*Sander lucioperca* (KP125333)		*Sander vitreus* (MN853162)		*Echiichthys vipera* (MT410919)		*Trachinus draco* (AP006024)	
	**Count**	**RSCU**	**Count**	**RSCU**	**Count**	**RSCU**	**Count**	**RSCU**	**Count**	**RSCU**	**Count**	**RSCU**	**Count**	**RSCU**	**Count**	**RSCU**	**Count**	**RSCU**	**Count**	**RSCU**
CGG (R)	18	1.40	5	0.38	8	0.63	13	0.99	9	0.47	6	0.49	6	0.47	12	0.94	6	0.48	9	0.74
AUU (I)	142	1.22	172	1.41	173	1.44	176	1.48	181	1.36	191	1.52	193	1.55	160	1.29	192	1.54	167	1.46
AUC (I)	120	1.03	91	0.75	88	0.73	85	0.72	86	0.64	78	0.62	77	0.62	100	0.81	81	0.65	85	0.74
AUA (M)	87	0.75	103	0.84	99	0.83	95	0.80	91	1.24	107	0.85	103	0.83	112	0.90	102	0.82	92	0.80
AUG (M)	66	1.0	47	1.00	48	1.00	55	1.00	56	0.76	47	1.00	45	1.00	44	1.00	58	1.0	60	1.00
ACU (T)	48	0.63	60	0.82	56	0.76	61	0.82	60	0.79	60	0.83	60	0.82	68	0.93	80	1.02	68	0.90
ACC (T)	130	1.72	118	1.61	120	1.63	119	1.60	126	1.66	116	1.61	114	1.56	100	1.37	98	1.24	115	1.53
ACA (T)	113	1.49	103	1.40	104	1.41	97	1.31	110	1.45	98	1.36	104	1.42	106	1.46	122	1.55	106	1.41
ACG (T)	12	0.16	13	0.18	15	0.20	20	0.27	7	0.09	14	0.19	14	0.19	17	0.23	15	0.19	12	0.16
AAU (N)	42	0.74	43	0.75	45	0.78	42	0.73	39	0.67	54	0.93	55	0.95	55	0.96	56	0.94	42	0.68
AAC (N)	71	1.26	72	1.25	70	1.22	73	1.27	77	1.33	62	1.07	61	1.05	60	1.04	63	1.06	82	1.32
AAA (K)	65	1.76	67	1.81	67	1.79	58	1.55	66	1.76	71	1.92	72	1.92	65	1.73	55	1.51	58	1.55
AAG (K)	9	0.24	7	0.19	8	0.21	17	0.45	9	0.24	3	0.08	3	0.08	10	0.27	18	0.49	17	0.45
AGU (S)	11	0.28	19	0.49	17	0.43	16	0.41	14	0.36	19	0.47	17	0.43	21	0.51	18	0.44	13	0.32
AGC (S)	46	1.15	36	0.92	38	0.97	40	1.02	40	1.03	36	0.89	37	0.93	38	0.93	46	1.12	48	1.19
AGA (R)	0	0	0	0	0	0	0	0	0	0	0	0	0	0	0	0	0	0	0	0
AGG (R)	0	0	0	0	0	0	0	0	0	0	0	0	0	0	0	0	0	0	0	0
GUU (V)	65	1.18	75	1.30	88	1.53	82	1.41	80	1.45	72	1.33	74	1.35	70	1.20	66	1.25	74	1.23
GUC (V)	68	1.23	59	1.03	61	1.06	59	1.02	49	0.89	52	0.96	53	0.97	67	1.15	39	0.74	61	1.02
GUA (V)	62	1.12	75	1.30	64	1.11	66	1.14	71	1.29	67	1.24	65	1.19	73	1.25	85	1.60	81	1.35
GUG (V)	26	0.47	21	0.37	17	0.30	25	0.43	20	0.36	26	0.48	27	0.49	24	0.41	22	0.42	24	0.40
GCU (A)	50	0.56	87	0.98	77	0.86	83	0.94	79	0.89	69	0.77	67	0.75	85	0.95	85	1.03	71	0.81
GCC (A)	180	2.0	147	1.65	158	1.77	47	1.66	143	1.62	158	1.77	162	1.81	149	1.67	133	1.61	142	1.62
GCA (A)	111	1.23	111	1.25	105	1.17	106	1.20	120	1.36	122	1.37	123	1.37	110	1.23	105	1.27	118	1.35
GCG (A)	19	0.21	11	0.12	18	0.20	18	0.20	12	0.14	8	0.09	7	0.08	13	0.15	8	0.10	19	0.22
GAU (D)	18	0.47	34	0.86	25	0.65	33	0.84	28	0.74	28	0.73	26	0..68	27	0.72	32	0.88	28	0.78
GAC (D)	59	1.53	45	1.14	52	1.35	46	1.16	48	1.26	49	1.27	51	1.32	48	1.28	41	1.12	44	1.22
GAA (E)	80	1.57	75	1.50	71	1.42	74	1.48	71	1.42	70	1.40	73	1.46	73	1.45	65	1.38	69	1.42
GAG (E)	22	0.43	25	0.50	29	0.58	26	0.52	29	0.58	30	0.60	27	0.54	28	0.55	29	0.62	28	0.58
GGU (G)	44	0.72	46	0.75	47	0.77	58	0.95	37	0.60	61	0.99	60	0.97	38	0.61	46	0.78	40	0.65
GGC (G)	77	1.26	77	1.26	77	1.26	74	1.21	80	1.30	69	1.12	71	1.15	80	1.29	77	1.31	85	1.39
GGA (G)	80	1.31	78	1.28	72	1.18	61	1.00	85	1.38	77	1.25	75	1.21	72	1.16	52	0.89	71	1.16
GGG (G)	43	0.70	43	0.70	49	0.80	51	0.84	44	0.72	39	0.63	41	0.66	58	0.94	60	1.02	49	0.80
Total	3800	62.00	3800	62.01	3800	61.45	3800	62.02	3799	60.00	3799	61.95	3800	61.33	3800	61.99	3798	62.02	3805	62.00

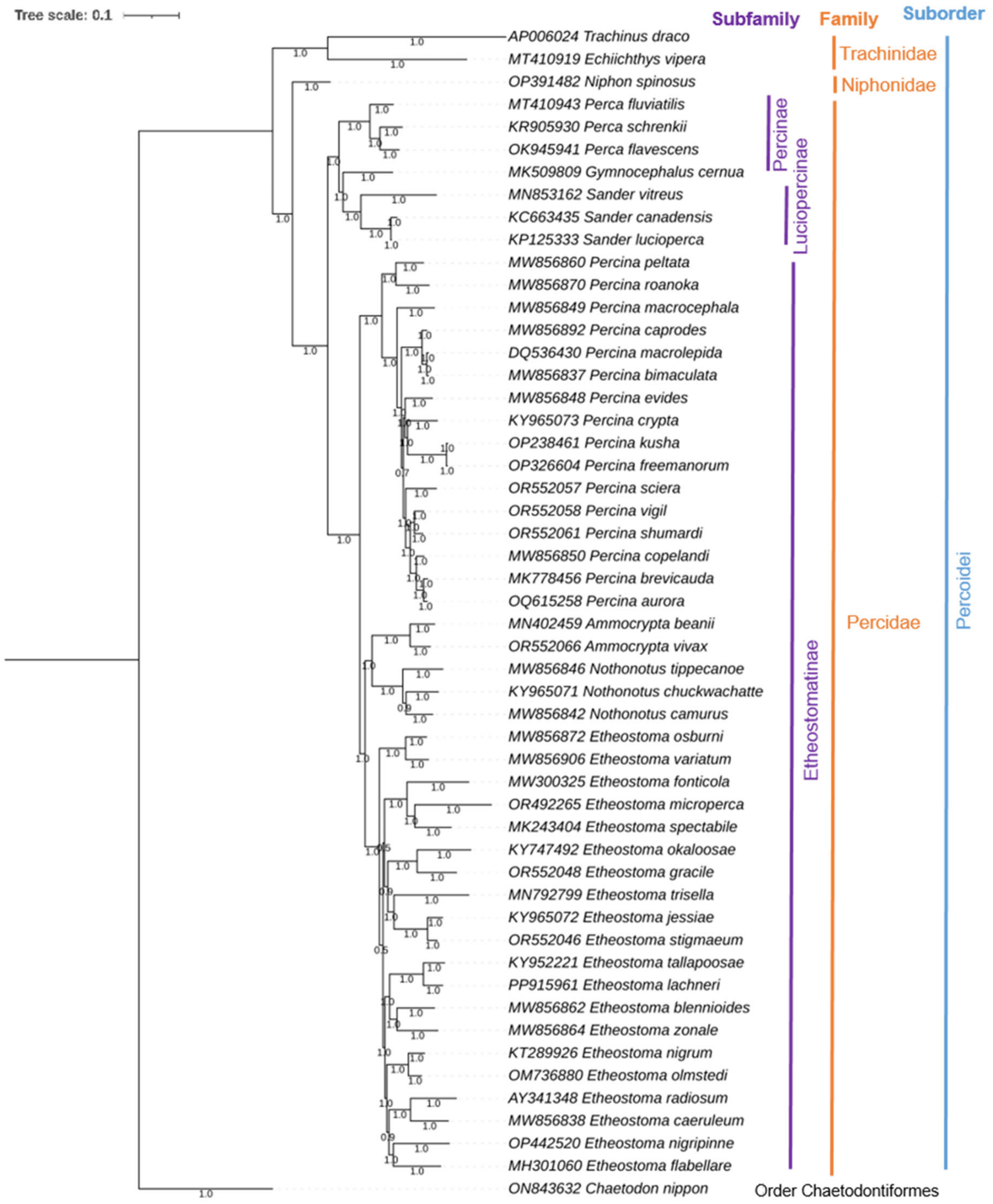

Figure S1. The Bayesian phylogeny constructed from 13 concatenated PCGs clearly distinguishes *Niphon spinosus* from other species of Percoidei. Bayesian posterior probability support values are indicated at each node, reflecting the statistical support for each branching point in the tree.
